# Characterizing Extracellular Vesicles Generated from the Integra CELLine Culture System and Their Endocytic Pathways for Intracellular Drug Delivery

**DOI:** 10.3390/pharmaceutics16091206

**Published:** 2024-09-13

**Authors:** Tianjiao Geng, Lei Tian, Song Yee Paek, Euphemia Leung, Lawrence W. Chamley, Zimei Wu

**Affiliations:** 1 School of Pharmacy, Faculty of Medical and Health Sciences, University of Auckland, Auckland 1010, New Zealand; t.geng@auckland.ac.nz (T.G.); lei.tian@auckland.ac.nz (L.T.); 2 Department of Pharmacy, Renji Hospital, Shanghai Jiao Tong University School of Medicine, Shanghai 200127, China; 3 Department of Obstetrics and Gynaecology, Hub for Extracellular Vesicles Investigations, Faculty of Medical and Health Sciences, University of Auckland, Auckland 1010, New Zealand; s.paek@auckland.ac.nz (S.Y.P.); l.chamley@auckland.ac.nz (L.W.C.); 4 Auckland Cancer Society Research Centre, Faculty of Medical and Health Sciences, University of Auckland, Auckland 1010, New Zealand; e.leung@auckland.ac.nz

**Keywords:** small extracellular vesicles, large extracellular vesicles, cellular uptake, endocytic pathways, size, extracellular vesicles yield

## Abstract

Extracellular vesicles (EVs) have attracted great attention as promising intracellular drug delivery carriers. While the endocytic pathways of small EVs (sEVs, <200 nm) have been reported, there is limited understanding of large EVs (lEVs, >200 nm), despite their potential applications for drug delivery. Additionally, the low yield of EVs during isolation remains a major challenge in their application. Herein, we aimed to compare the endocytic pathways of sEVs and lEVs using MIA PaCa-2 pancreatic cancer cell-derived EVs as models and to explore the efficiency of their production. The cellular uptake of EVs by MIA PaCa-2 cells was assessed and the pathways were investigated with the aid of endocytic inhibitors. The yield and protein content of sEVs and lEVs from the Integra CELLine culture system and the conventional flasks were compared. Our findings revealed that both sEVs and lEVs produced by the Integra CELLine system entered their parental cells via multiple routes, including caveolin-mediated endocytosis, clathrin-mediated endocytosis, and actin-dependent phagocytosis or macropinocytosis. Notably, caveolin- and clathrin-mediated endocytosis were more prominent in the uptake of sEVs, while actin-dependent phagocytosis and macropinocytosis were significant for both sEVs and lEVs. Compared with conventional flasks, the Integra CELLine system demonstrated a 9-fold increase in sEVs yield and a 6.5-fold increase in lEVs yield, along with 3- to 4-fold higher protein content per 10^10^ EVs. Given that different endocytic pathways led to distinct intracellular trafficking routes, this study highlights the unique potentials of sEVs and lEVs for intracellular cargo delivery. The Integra CELLine proves to be a highly productive and cost-effective system for generating EVs with favourable properties for drug delivery.

## 1. Introduction

Extracellular vesicles (EVs) are cell-secreted heterogeneous lipid bilayer structures and play important roles in transporting biological information and materials among cells, both locally and over long distances [1]. As such, the potential usage of EVs as drug delivery carriers, including small EVs (sEVs, <200 nm) [2,3,4,5] and large EVs (lEVs, >200 nm) [6,7], as defined by the International Society for Extracellular Vesicles in 2018 [8], has been extensively investigated in recent years. Both sEVs and lEVs are highly heterogeneous in size and surface proteins due to their different biogenesis pathways: sEVs are mainly composed of exosomes, which are derived from the multivesicular bodies (MVBs), whereas lEVs are mainly derived from the plasma membrane [8]. Encouraging proof-of-concept studies suggested that EVs are promising therapeutic carriers for the treatment of immunological disorders [9], neurological disorders [10], and cancers [11]. The efficiency of drug delivery depends on the internalization mechanism by recipient cells and the subsequent intracellular trafficking. Various cellular uptake pathways might affect the kinetics of EVs’ intracellular trafficking and the final fate of EVs’ cargoes [12]. In our previous study, it was found the majority of sEVs were prone to being entrapped in the endosomes, limiting the cellular bioavailability of drugs [13]. Therefore, understanding the cell entry and intracellular trafficking mechanisms of EVs is of vital importance for accessing the fate of EVs and their potentials as drug delivery vehicles.

EVs have been reported to enter cells via different pathways, including membrane fusion, receptor interaction, and endocytosis, and EVs may gain cell entry via more than one route [14]. Objects with different sizes and physical and biological parameters may undergo endocytosis by two main pathways: phagocytosis (cell eating) and pinocytosis (cell drinking) [15,16]. Phagocytosis involves the internalization of relatively large particles (>0.5 μm) and is typically restricted to specialised professional phagocytes [17]. Pinocytosis, in contrast, occurs in all types of cells and can be further subdivided into macropinocytosis (fluids and micro-sized particles > 1 μm [18]), clathrin-mediated endocytosis (particles around 100–500 nm) [19], clathrin- and caveolin-independent endocytosis (particles around 90 nm) [20,21], and caveolin-mediated endocytosis (particles around 60–80 nm) [22]. Vesicles will internalize in specific intracellular regions via different pathways [23]. 

Recent studies have investigated the endocytic mechanisms of EVs, but the results have varied. For example, Verdera et al. showed that EVs (60–250 nm, with the majority around 100 nm) derived from human epidermoid carcinoma A431 cells entered HeLa cells by both clathrin-independent endocytosis and macropinocytosis [24]. Another study showed that caveola-mediated endocytosis was involved in the cellular uptake of exosomes (size was not reported) derived from Epstein–Barr virus-infected B cells to cellosaurus cells CNE1 [25]. On the other hand, lEVs are also used as intracellular delivery platforms, particularly in cancer treatment [6,26,27]. The larger size of lEVs compared to sEVs enables them to have higher drug loading and capacity to encapsulate macromolecular drugs or even drug-containing nanoparticles. Additionally, lEVs are produced directly through outward budding of the cell membrane and their surface protein composition is largely similar to parental cells, which facilitate their targeted delivery ability to recipient cells. Indeed, Xu et al. reported that lEVs derived from epithelial cells could serve as a safe and effective drug delivery carriers for CRISPR/Cas9 to xenograft mouse models [27]. Saari et al. encapsulated the small molecule drug Paclitaxel in cancer cell-derived lEVs and delivered the drug to their autologous cancer cells effectively [6]. However, like many other studies, there is limited information on the detailed endocytic pathways of lEVs. Therefore, understanding whether different EVs subpopulations from the same cell of origin are processed through similar mechanisms is of great interest. 

Herein, this study aimed to understand the endocytic routes involved in sEVs and lEVs with their donor cells using a human pancreatic cancer cell line, MIA PaCa-2, as an EVs source. Cancer cell-derived EVs have tumour-homing and immune-stimulating abilities, which strengthens their conceptual use as an ideal source of delivery vehicles for tumour-targeting [28]. Following characterization of EVs, including the size, morphology, and protein content, the cellular uptake of PKH67-labelled sEVs or lEVs was compared by fluorescence microscopy, and the endocytic pathways were differentiated by the use of the inhibitors. A series of pharmacological inhibitors that selectively inhibit different endocytic pathways were applied, including genistein, chlorpromazine (CPZ), and cytochalasin D (CytoD) for caveolin-mediated endocytosis, clathrin-mediated endocytosis, and actin-dependent phagocytosis or macropinocytosis, respectively. 

Additionally, one of the major challenges limiting the application of EVs is their low yield during isolation [29]. In this study, a new Integra CELLine culture system was used to generate EVs, and the total particle number and protein content of the EVs were compared with those from the conventional flasks. EVs produced by the Integra CELLine culture system were used for investigation of endocytic pathways. 

## 2. Materials and Methods

### 2.1. Reagents

Reagents for cell culture, Hoechst 33342, paraformaldehyde, Citifluor Mountant Solution AF1, phosphate-buffered saline (PBS, pH 7.4), and Pierce^TM^ BCA protein assay kit were purchased from Thermo Fisher Scientific (Auckland, New Zealand). Endocytic inhibitors (genistein, Cat. No. G6649, chlorpromazine hydrochloride, Cat. No. C8138, cytochalasin D, Cat. No. C8273), thiazolyl blue tetrazolium bromide, PKH67 dye, the Integra CELLine AD (adhere) 1000 flask (CLAD1000), dimethyl sulfoxide (DMSO), and Corning^®^ tissue-culture treated culture dishes (D × H 35 mm × 10 mm) were all obtained from Sigma-Aldrich Ltd. (Auckland, New Zealand). Chemically Defined Medium For High Density Cell Culture (CDM-HD) was obtained from FiberCell Laboratories (New Market, MD, USA). Sulfo-cyanine5 N-hydroxysuccinamie (NHS) ester was purchased from Lumiprobe (Cockeysville, MD, USA). The pancreatic cancer MIA PaCa-2 cell line was a gift from the Auckland Cancer Society Research Centre, the University of Auckland.

### 2.2. Cell Culture and EVs Isolation

**Figure 1:**
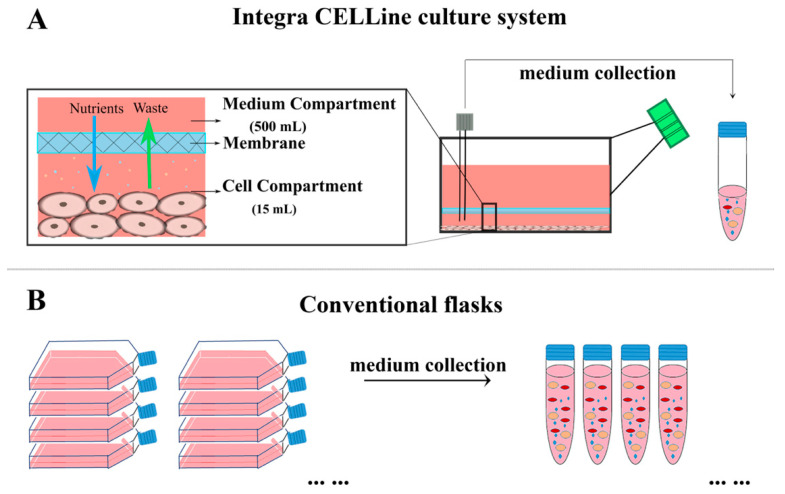
Schematic illustration of the Integra CELLine culture system (**A**) and conventional flasks (**B**) and the set-up to generate EVs. With the Integra CELLine culture system, cells can be continuously used for up to 300 days while medium containing EVs was collected twice per week. However, conventional flasks only can be used for one week and medium containing EVs was collected once.

The Integra CELLine culture system to generate EVs alongside the conventional method using flasks are illustrated in Figure 1.

#### 2.2.1. Cell Culture Using the Integra CELLine Culture System

Immortalized MIA PaCa-2 cells were expanded in conventional culture flasks and seeded at an initial density of 2 × 10^7^ cells in the Integra CELLine culture system (Integra Biosciences AG, Hudson, NH, USA). Cells were adapted for one week to culture conditions by gradually changing the culture medium to Advanced Dulbecco’s Modified Eagle’s Medium/Nutrient Mixture F-12 (DMEM/F12) containing 2% (*v*/*v*) CDM-HD protein-free supplement and 1% (*v*/*v*) penicillin/streptomycin (PS). Medium in the cell compartment containing EVs (15 mL) was harvested twice per week, and the cell compartment was washed with PBS to remove any non-adherent or dead cell/debris before adding fresh medium. The outer “nutrient” chamber was filled with 500 mL DMEM containing 2% (*v*/*v*) fetal bovine serum (FBS) and 1% (*v*/*v*) PS. This design allows high-density cell cultures by providing a continuous supply of nutrients. Cells can be used without replacement for more than 300 days [30].

#### 2.2.2. Cell Culture Using Conventional Flasks

MIA PaCa-2 cells were maintained in multiple conventional 175 cm^2^ flasks with DMEM containing 10% (*v*/*v*) and 1% (*v*/*v*) PS in an atmosphere of 95% humidity and 5% CO_2_ at 37 °C. To avoid EVs contamination from FBS, cells were grown in DMEM containing 10% (*v*/*v*) FBS for 2 days for cell attachment. After that, cells were cultured in serum-free DMEM for 2 days before EVs isolation.

#### 2.2.3. Methods for EVs Isolation

EVs from the cell culture medium, either from the Integra CELLine system or the conventional flasks, were collected and purified by the standard differential ultracentrifugation method [8]. The medium was first centrifuged at 2000× *g* for 5 min at 4 °C to pellet the cellular debris. The supernatant was then ultracentrifuged at 20,000× *g* for 1 h at 4 °C to obtain the enriched lEVs. The supernatant was further ultracentrifuged at 100,000× *g* for 1 h at 4 °C to pellet the enriched sEVs (Avanti J30I Ultracentrifuge, JA 30.50 fixed angle rotor, Beckman Coulter, Brea, CA, USA). Both lEVs and sEVs enriched pellets were resuspended in PBS and stored at 4 °C for the downstream studies.

### 2.3. Characterization of EVs

Immediately after isolation, the yield, size, and morphology were characterized, and the protein contents of sEVs and lEVs from the Integra CELLine culture system were compared with those from conventional flasks as shown below. 

#### 2.3.1. Evaluation of the EVs Yield

The EVs yield was evaluated by calculating two indicators in the same amount of culture medium: (1) the EVs number, and (2) the EVs protein quantification. 

#### 2.3.2. Nanoparticle Tracking Analysis (NTA)

The size and concentration (particle number/mL) of sEVs and lEVs from the Integra CELLine culture system were assessed by NTA using the NanoSight NS300 instrument (Malvern Panalytical Ltd., Malvern, UK) equipped with a 405 nm laser and an sCMOS camera. Experimentally, sEVs and lEVs were diluted with PBS to reach a measurable concentration range of 10^7^–10^9^ particles/mL. A single measurement consists of three 30 s videos with 10 s delays in between, and each sample was measured three times at cameral level 10. The detection threshold was set at 10, and data were acquired and analysed on the NTA software 3.0 in real time by associating Brownian motion with particle size. Only measurements with over 1000 valid tracks/vesicles were included in the analysis.

#### 2.3.3. Dynamic Light Scattering (DLS)

To determine the size, polydispersity index (PDI), and zeta potential of sEVs and lEVs, sEVs and lEVs were diluted in PBS as appropriate before DLS analysis using the Zetasizer Nano ZS (Malvern Panalytical Ltd., Malvern, UK), and 10 spectra were recorded for each sample at 25 °C. Data were obtained from three replicates.

#### 2.3.4. Cryogenic Transmission Electron Microscopy (Cryo-TEM)

To visualize the ultrastructural morphology of sEVs and lEVs, cryogenic transmission electron microscopy (cryo-TEM) was used. Samples (10 μL, 2 × 10^11^ particles/mL) were transferred to a chamber by placing a drop onto a copper grid. The sample was then blotted to leave a thin film stretched over grid holes, and the grid was dipped into liquid ethane and cooled to 90 K with liquid nitrogen. Samples were observed on a Tecnai 12 electron microscope (FEI, Hillsboro, OR, USA) operating at 120 kV to study the EVs’ morphology and structures. 

#### 2.3.5. Total Protein Content by Bicinchoninic Acid (BCA) Assay

The protein content of sEVs and lEVs was quantified by the BCA assay following the manufacturer’s instructions. First, bovine serum albumin (BSA) was dissolved in 1× Radioimmunoprecipitation assay buffer (RIPA buffer) to make stock solutions of 25 μg/mL to 2000 μg/mL. Each sample and standards (10 μL) in triplicates were pipetted into a 96-well plate. Then, BCA Working Reagent (200 μL, 50:1, Reagent A:B) was added to each well. The plate was covered and incubated at 37 °C for 30 min. After that, samples and standards were cooled to room temperature and measured on an xMark spectrophotometer at 562 nm. A standard curve of the absorbance against the BCA concentration was constructed and used to calculate the protein concentrations in the samples.

### 2.4. Evaluation of the Cellular Uptake of EVs 

Fluorescent dye-labelled sEVs and lEVs from the Integra CELLine culture system were evaluated on MIA PaCa-2 cells using a fluorescence plate reader over 3 h observation for their kinetic cellular uptake. The cellular uptake was also visualized by fluorescence microscopy, and the fluorescence intensity was quantified by Fiji software (https://imagej.net/software/fiji/downloads, accessed on 5 September 2024).

#### 2.4.1. The Kinetics of Cellular Uptake of EVs

**Figure 2:**
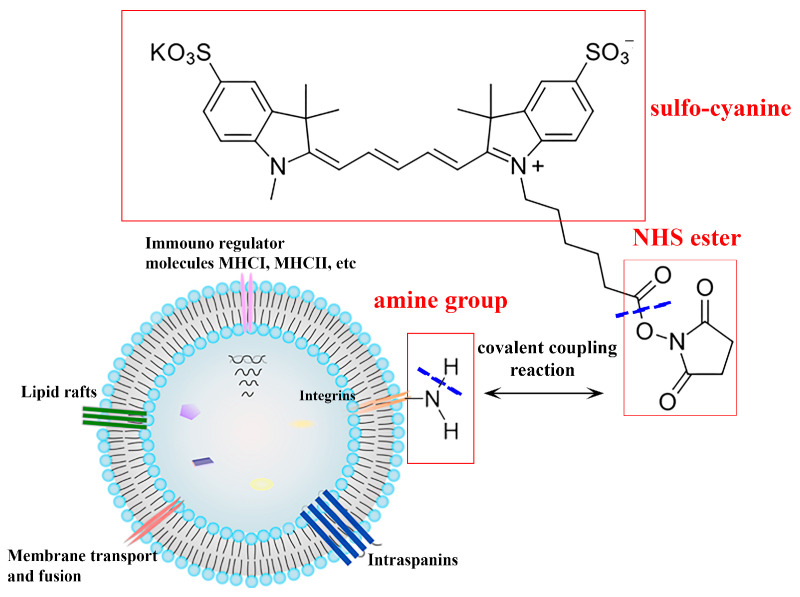
The structure of sulfo-cyanine5 NHS ester (Cy5) (MW 777.49 g/mol), a red fluorescent dye for EVs labelling. Labelling of EVs was achieved through covalent coupling between NHS ester groups and amine groups on proteins within the EVs. Excitation maximum ~646 nm; emission maximum ~662 nm.

MIA PaCa-2 cells were seeded in a 96-well plate at a density of 5 × 10^3^ cells/well in 100 μL medium and cultured for 24 h. The EVs were pre-labelled with a red/near-infrared fluorescent dye sulfo-cyanine5 NHS ester (Cy5) for their kinetic cellular uptake study. The reaction is shown in Figure 2. sEVs or lEVs (2 × 10^10^) in 100 μL PBS were incubated with l μL Cy5 (100 μg/mL) at 37 °C for 2 h in darkness with gentle shaking at 500 rpm using an Eppendorf ThermoMixer^®^ C (Eppendorf, Hamburg, Germany). Then, the free dye was removed and washed with PBS by 1 h ultracentrifugation at 20,000 × *g* for lEVs and 100,000 × *g* for sEVs at 4 °C, respectively. The pellets were resuspended in 100 μL PBS and stored at 4 °C for downstream applications.

Next, the Cy5-labelled sEVs or lEVs were incubated with cells (4 × 10^4^ EVs/cell) at 37 °C for 5, 15, 30, 45, 60, 120, and 180 min, respectively. Thereafter, free sEVs or lEVs remaining in the medium were removed by washing the cells with 200 μL PBS. The fluorescence intensity (FI) of the internalized sEVs/lEVs was then measured in a microplate reader (λex/λem: 646/662 nm, 2300 Enspire, Perkin Elmer, Hopkinton, MA, USA). Autofluorescence background reading from untreated control cells was deducted for kinetic analysis.

#### 2.4.2. Quantification of Cellular Uptake of EVs

To track the cellular uptake of EVs, the EVs were first fluorescently labelled with PKH67 lipid dye. The EVs pellet was mixed with 1 μL of PKH67 prepared in 500 μL diluent C solution (a solvent used for general membrane labelling). The sample was gently agitated for 30 s and incubated for 5 min at room temperature according to the manufacturer’s protocol. Free dye was removed by 1 h ultracentrifugation at 20,000× *g* for lEVs and 100,000 × *g* for sEVs at 4 °C, respectively. Then, the samples were washed with PBS followed by another 1 h ultracentrifugation. Finally, PKH67-labelled EVs pellets were resuspended in PBS (0.1 M, pH 7.4) and stored at 4 °C for downstream cellular uptake study.

MIA PaCa-2 cells (2 × 10^5^) were seeded in a cell culture Petri dish (D × H 35 mm × 10 mm) with one coverslip and cultured for 24 h. After that, PKH67-labelled sEVs or lEVs were added to cells (4 × 10^4^ EVs/cell) and incubated for 1 h or 2 h, respectively. The nucleus was then stained with Hoechst 33342 at 37 °C for 20 min, and cells were washed with PBS three times after treatment, followed by fixation in 4% paraformaldehyde solution for 15 min at room temperature. Cells were washed with PBS after fixing. The coverslips bearing the MIA PaCa-2 cells were then removed from the culture dish and placed on another slide with the Citifluor Mountant Solution. Images were taken from a fluorescent microscope (Nikon Eclipse E400, Nikon Metrology Inc., Brighton, MI, USA). Mean FI from individual cells was calculated by Fiji software.

### 2.5. Investigation of EVs Endocytic Pathways

#### 2.5.1. Optimization of Inhibitor Pre-Treatment Concentration

The internalization mechanisms of EVs were investigated using the methods reported previously [31,32]. The optimal concentration of each inhibitor was firstly determined. MIA PaCa-2 cells (5 × 10^3^/well) in 100 μL medium were seeded in a 96-well plate and pre-incubated with genistein (initially dissolved in DMSO) at a final concentration of 25, 50, and 75 μg/mL [33], respectively. After 3 h, cell viability was observed under the microscope (EVOS™ XL Core Imaging System, Thermo Fisher Scientific). Similarly, CPZ or CytoD (dissolved in PBS pH 7.4) was added to each well, with the final concentration falling in the range of 5–15 μg/mL [33,34,35,36]. To test the cell viability, the cell culture medium was aspirated, and 200 μL of fresh medium containing MTT solution (5 mg/mL) was added to each well. The plate was incubated at 37 °C for 4 h, and the medium was replaced with 100 μL of DMSO to dissolve the formazan. The plate was read at 570 nm with a microplate reader (Varioskan^TM^ LUX, Thermo Fisher Scientific, Auckland, New Zealand). The drug concentration causing 50% inhibition of the cell viability (IC_50_) was calculated using a non-linear fitting model in GraphPad Prism 8.2 (GraphPad Software, San Diego, CA, USA).

#### 2.5.2. Determination of Endocytic Pathways of sEVs and lEVs

MIA PaCa-2 cells (2 × 10^5^) were seeded in a cell culture Petri dish with one coverslip. After cultured for 24 h, cells were pre-treated with inhibitors at 37 °C for 1 h, and medium-only-treated cells were used as controls. Then, PKH67-labelled sEVs or lEVs were added to cells (4 × 10^4^ EVs/cell) and incubated for 1 h or 2 h. The nucleus was stained with Hoechst 33342 at 37 °C for 20 min, and cells were washed with PBS three times before the fixation in 4% paraformaldehyde solution for 15 min at room temperature. Cells were washed once with PBS. The coverslips bearing the MIA PaCa-2 cells were then removed from the culture dish and placed on another slide with the Citifluor Mountant Solution. All experiments were performed in triplicates. Images were taken from the fluorescent microscope (Nikon Eclipse E400). FI was calculated by Fiji software. The cellular uptake (%) was expressed by the following equation to evaluate the inhibitor effect:

$$Cellular\;uptake\left(\%\right)=\frac{Fluorescence\;of\;EVs\;taken\;up\;by\;cells\;with\;inhibitors}{Fluorescence\;of\;EVs\;taken\;up\;by\;cells\;without\;inhibitors}\times 100$$

### 2.6. Data Analysis 

All data are presented as mean ± standard deviation (SD). Comparison of the means between groups was using one-way ANOVA with GraphPad Prism 8.2 software, and differences in *p*-values less than 0.05 were considered statistically significant.

## 3. Results and Discussion

### 3.1. Evaluation of EVs Yield

The Integra CELLine culture system was introduced in 2008 to obtain high quantities of exosomes for both adherent and suspension cell lines at a low cost and in less time for the users [30]. However, reports in the literature [30,37] have only measured exosome yield by measuring the particle number or protein content. In this study, the yield of sEVs and lEVs were compared by measuring both particle numbers and protein contents for a better understanding. For reference, the EVs yield was also compared with the method using conventional 175 mL flasks. In addition, the Western blotting analysis showed the presence of sEVs markers (CD81 or TSG101) and confirmed the successful isolation of sEVs in our previous studies [13,38]. Western blotting analysis of lEVs was not conducted. Based on their biogenesis, lEVs usually have the similar biomolecules expression to their donor cell membranes [39]. 

#### 3.1.1. EVs Yield by Measuring EVs Number 

**Figure 3:**
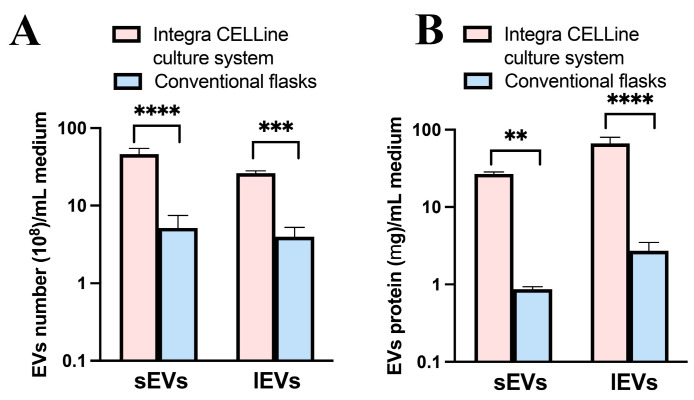
The EVs yield from the Integra CELLine culture system compared with that from conventional flasks. Data are expressed as EVs number per mL medium (**A**) and EVs protein (μg) per mL medium (**B**) (mean ± SD, *n* = 3). ** *p* < 0.01, *** *p* < 0.005, **** *p* < 0.001.

It was found that with the same volume of cell culture medium used, the Integra CELLine culture system produced approximately 9.0-fold and 6.5-fold more sEV and lEV particles compared to conventional flasks, respectively (Figure 3A). This is similar to a 10-fold difference in the number of sEVs (60–140 nm) that were derived from three different types of cells—human oral squamous cell carcinoma PE/CA-PH49/E10, pancreatic adenocarcinoma BxPC3, and human melanoma brain metastasis H3 cell lines [37]. 

#### 3.1.2. EVs Yield by Measuring EVs Protein Content 

The protein content of EVs derived from MIA PaCa-2 cells using conventional flasks was similar to that reported previously [40]. Compared with this, significantly higher EVs protein content per mL of medium was obtained from the Integra CELLine culture system (30.4-fold sEVs and 35.1-fold lEVs, respectively) (Figure 3B). This can be explained by the fact that the protein content per 10^10^ sEVs or lEVs from the Integra CELLine culture system was 3- and 4-fold greater than those from the conventional flasks, respectively (60.0 ± 13.0 μg vs. 19.0 ± 8.8 μg for sEVs, 258.2 ± 72.0 μg vs. 69.8 ± 17.6 μg for lEVs). This is probably because the small proteins in the medium could flow through the 10 kDa membrane into the compartment that cultured EVs, resulting in impurity of the EVs [37]. Furthermore, the ultracentrifugation-based protocol for EVs purification used in this study may contribute to the high amount of protein, as this approach can co-isolate a complex assortment of non-vesicular materials [41]. 

The development of a EVs delivery platform requires large quantities of EVs. To increase EVs yield using conventional flasks, scaling up the number of flasks is the only option, but it is time-consuming, costly, and requires the handling of high volumes of cell culture medium. Moreover, the large volume of the cell culture medium is limited by the maximum capacity (volume of cell culture medium) of the ultracentrifuge being used. This represents a significant practical issue hampering progress in this research area [30]. 

In contrast, the Integra CELLine culture system enables the maintenance of large numbers of cells (maximum 400 million) in a medium volume of 15 mL due to its unique characteristics [30]. This is a two-compartment culture system with a cell compartment designed for cells to sustain cell growth at high densities and a medium compartment where a cell-free culture medium is placed. The cell and medium compartments are separated by a semi-permeable cellulose acetate membrane with 10 kDa pores which allows a continuous exchange of nutrients and waste. A woven mesh inside the cell compartment provides cells with support for adherence and growth, and a silicone membrane at the bottom allows for direct oxygenation and gas exchange [37]. 

Compared with conventional flasks, the Integra CELLine culture system represents a superior method for achieving a high EVs yield: (1) using a small volume of culture medium for ultracentrifugation; (2) less frequent EVs collection (twice a week) without the need to replace cells for over a period of approximately 300 days [42]; (3) no need to change the medium to a serum-free medium before EVs isolation because a CDM-HD protein-free supplement can be used in the cell compartment. 

### 3.2. Characterization of EVs from the Integra CELLine Culture System 

#### 3.2.1. Size Distribution of EVs by NTA 

**Figure 4:**
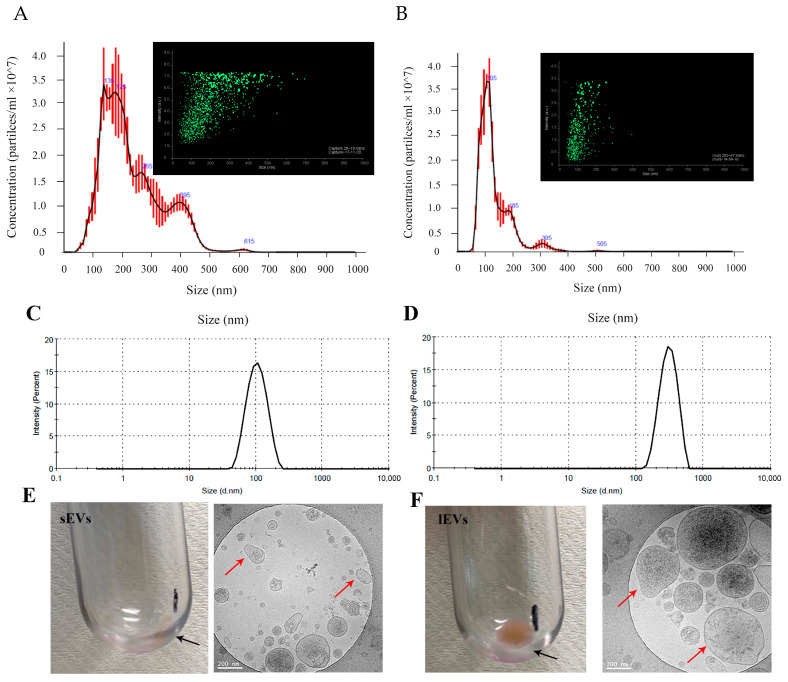
Characterisation of EVs isolated from the Integra CELLine culture system. Nanoparticle tracking analysis (NTA) measurement of sEVs (**A**) and lEVs (**B**) isolated from MIA PaCa-2 cells. Size distribution of sEVs (**C**) and lEVs (**D**) measured by dynamic light scattering (DLS). Representative EVs samples in tubes (black arrows) and cryogenic transmission electron microscopic (cryo-TEM) micrographs of sEVs (**E**) and lEVs (red arrows) (**F**). Scale bars: 200 nm.

NTA can measure particles from 30 to 1000 nm using laser light scattering microscopy with a charge-coupled device camera [43]. As shown in Figure 4A,B, sEVs from the Integra CELLine culture system had an average size of 125.5 ± 0.6 nm, peaking at 105 nm (Mode size or the major peak) and ranging from 50 to 400 nm. The majority of them (95%) had a size less than 200 nm (50% < 100 nm), and 5% of them were over 200 nm (1% > 500 nm). These results are similar to the reported data [44]. In contrast, lEVs had a broader distribution, with an average size of 222.7 ± 6.4 nm, peaking at 145 nm. The majority of the lEVs had a size ranging from 100 nm to 500 nm, with only 3% over 500 nm and 10% below 100 nm. The latter overlapped with the size of sEVs. This probably resulted from the differential ultracentrifugation method we used, which is based on Stokes’ law, to separate EVs in terms of EVs density and size differences [29]. 

Particles with a size of 10–200 nm are generally considered suitable for cancer therapy as they can easily extravasate from the vascular leak of tumour vessels due to the enhanced permeability and retention effect (EPR effect) [45]. Both sEVs and lEVs are considered suitable candidates for anticancer drug delivery due to their ideal size. However, the large quantity of particles over 200 nm in lEVs might be a concern for intravenous administration.

#### 3.2.2. Size Distribution and Zeta Potential of EVs by DLS

The size distribution of sEVs and lEVs was also measured by DLS (Figure 4C,D). The vesicles historically designated as sEVs (<200 nm) were 106.5 ± 1.1 nm (PDI 0.28 ± 0.03), similar to the NTA results. The mean size of lEVs was 375.5 ± 7.0 nm (PDI 0.38 ± 0.02), relatively larger than the average size observed with NTA. In addition, DLS intensity did not show particles less than 100 nm as observed from NTA. This is because DLS results are biased towards larger particles in a heterogeneous mixture. The intensity of scattered light in DLS is proportional to the sixth power of the particle diameter, making smaller particles appear to be undetectable [46], especially when large particles exist.

The sEVs and lEVs both were negatively charged with a similar zeta potential (−28.1 ± 1.0 mV and −25.3 ± 1.2 mV). These are consistent with the reported values [47]. 

#### 3.2.3. Morphology of EVs by Cryo-TEM 

Cryo-TEM revealed that sEVs and lEVs from MIA PaCa-2 cells were heterogeneous in diameter and shape and displayed a spherical or oval-shaped appearance (Figure 4E,F). The majority of the sEVs were below 200 nm in diameter. However, lEVs contained large vesicles over 200 nm and small vesicles less than 100 nm, consistent with the NTA results. Interestingly, some of the lEVs were found to contain dense surface structure (the dark surface), while sEVs did not, which has also been reported in the literature [48]. We believe the difference in surface structure was attributed to the differences in the biogenesis pathways of sEVs (mainly exosomes) and lEVs (mainly microvesicles). lEVs adopt their parental cell’s glycocalyx while shedding from the cell’s plasma membrane, while sEVs are secreted from the cells through exocytosis, leading to different surface structures [48]. 

### 3.3. Evaluation of EVs Cellular Uptake

#### 3.3.1. The Kinetics of Cellular Uptake of sEVs and lEVs 

The Integra CELLine culture system generated EVs were effectively labelled with Cy5. Cy5 is a reactive dye for labelling amino-group in soluble proteins as well as in all kinds of peptides and oligonucleotides. Cyanine groups (fluorophores) bound with NHS ester can form a covalent bond with amine groups in EVs proteins [49]. 

**Figure 5:**
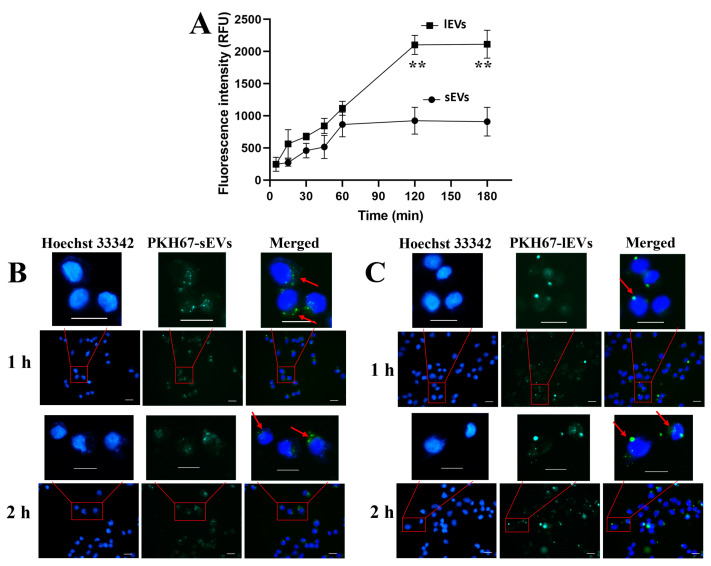
The cellular uptake of Cy5- or PKH67-labelled sEVs and lEVs by their donor MIA PaCa-2 cells. (**A**) Fluorescence intensity of Cy5 (relative fluorescence units, RFU) in sEVs and lEVs cellular uptake at different time points measured by a fluorescence plate reader. Fluorescence images of sEVs (**B**) and lEVs (**C**) labelled by PKH67 internalised by MIA PaCa-2 cells for 1 h and 2 h, respectively. Scale bars: 20 μm. Cells were exposed to the same number of EVs in both experiments. Data represent the means ± SD, and *n* = 3 cells each data point. ** *p* < 0.01. For clarity cells within the red boxed areas are highlighted in the upper panels and the arrows show that internalized EVs were prone to being entrapped in organelles.

The cellular uptake of both Cy5 labelled sEVs and lEVs by MIA PaCa-2 cells was found to be time-dependent (Figure 5A). For sEVs, at this treated concentration (4 × 10^4^ vesicles/cell), the cellular uptake reached the maximum by 1–2 h and plateaued after that. Interestingly, the cellular uptake of lEVs was increased until 2 h when it plateaued, possibly because particles with smaller size were preferentially taken up by cells [50,51]. The time-dependent uptake has also been observed by Franzen et al., who studied the exosomes derived from human bladder cancer SW780 cells on their donor cells [52]. It is worth noting that the FI of IEVs was similar compared with that of sEVs at 1 h but 2 times higher than sEVs at 2 h. This does not mean more lEVs enter cells than sEVs. The higher FI of lEVs could be explained by the observation that lEVs contained more proteins than sEVs and thus are likely to carry more Cy5 dye. 

#### 3.3.2. Quantification of Cellular Uptake of sEVs and lEVs

EVs were labelled with PKH67, a green fluorophores (excitation wavelength (λ_ex_) = 490 nm/emission wavelength (λ_em_) = 502 nm) with longer aliphatic carbon chains than other dyes of its family, facilitating intercalating into lipid membranes [53]. PKH67, along with other PKH dyes like PKH26, is extensively used for EVs labelling [54]. The cellular uptake of sEVs (Figure 5B) had an initial increase over time but no further change from 1 h to 2 h. In contrast, lEVs reached the maximal uptake at 2 h (Figure 5C). At the peak levels, the FI of lEVs-treated cells was significantly higher than that of the sEVs-treated cells (*p* < 0.01). This discrepancy is likely because the cellular uptake of the cargo was size-dependent, with smaller EVs potentially internalizing into cells more rapidly than lEVs [55]. However, due to the large loading capacity of lEVs, they elicited a stronger cellular FI than sEVs even cell death was observed at the equilibrium time (3 h). Additionally, the asymmetrical distribution of both sEVs and lEVs in cells was observed, likely due to entrapment in cellular organelles. We have previously demonstrated that endo/lysosomes were located on one side of the nuclei in MIA PaCa-2 cells [13], and natural sEVs are prone to being entrapped in these organelles following endocytosis. Additionally, large green dots were observed in cells treated with lEVs (Figure 5C). This observation persisted in subsequent endocytic studies, where the mechanisms of uptake were further explored. 

### 3.4. Comparison of Endocytic Pathways of sEVs and lEVs

#### 3.4.1. Optimization of Endocytic Inhibitors Concentration

Three kinds of endocytic inhibitors, including genistein, CPZ, and CytoD, were selected for probing contributions of each pathway as they are actively used in other studies investigating the endocytosis of EVs [24,31]. Their working mechanisms and commonly used concentration are shown in Table 1. 

**Figure 6:**
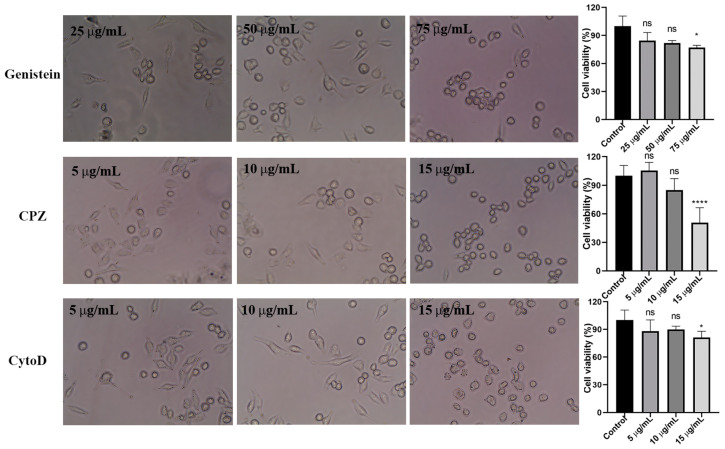
The effect of inhibitors concentrations on MIA PaCa-2 cells viability. Left: Images showing cell morphology after 3 h pre-treatment under light microscope (40×). Right: An MTT assay was carried out to evaluate the cell viability in the presence of inhibitors with different concentrations (mean ± SD, *n* = 3 batches). * *p* < 0.05, **** *p* < 0.001, ns = nonsignificant.

The cytotoxicity of the inhibitors at their commonly used concentrations were investigated to ensure the cell viability and function during the experiment. Figure 6 shows that cells pre-treated with inhibitors at a low or medium concentration had a similar morphology compared with cells without inhibitors. However, when further increasing the concentrations, most cells become round and detached. This was also observed in the MTT assay. Therefore, genistein at 50 μg/mL, CPZ at 10 μg/mL, and CytoD at 10 μg/mL were selected to pre-treat cells in the following pathway studies.

#### 3.4.2. Different Endocytic Pathways of sEVs and lEVs 

**Figure 7:**
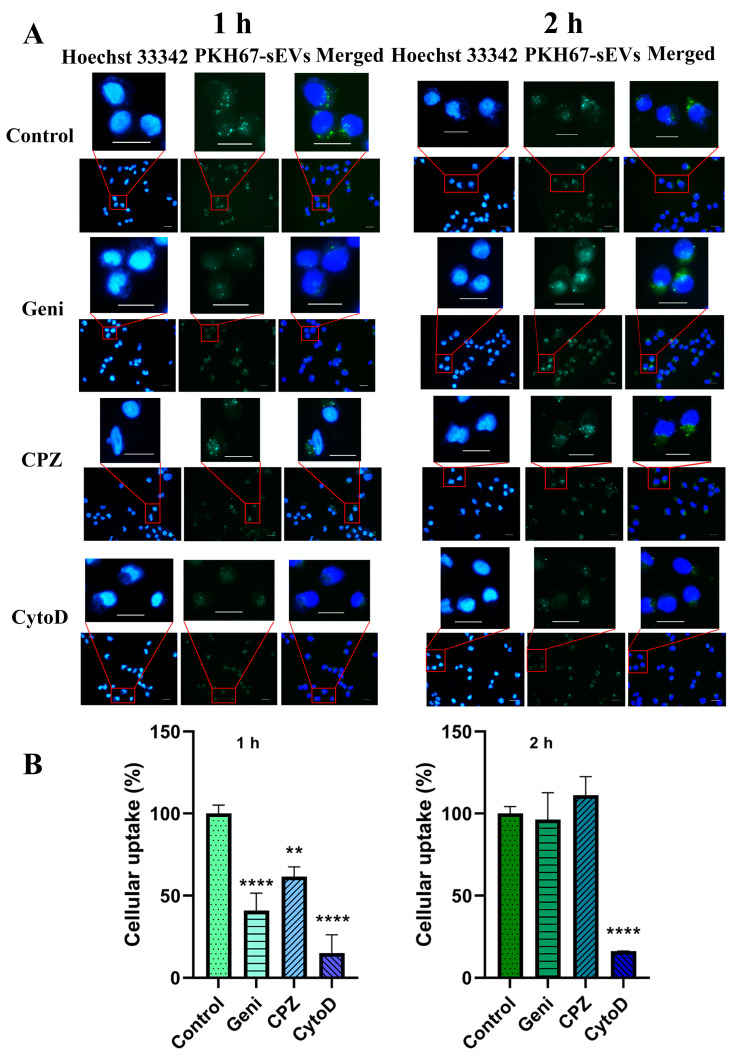

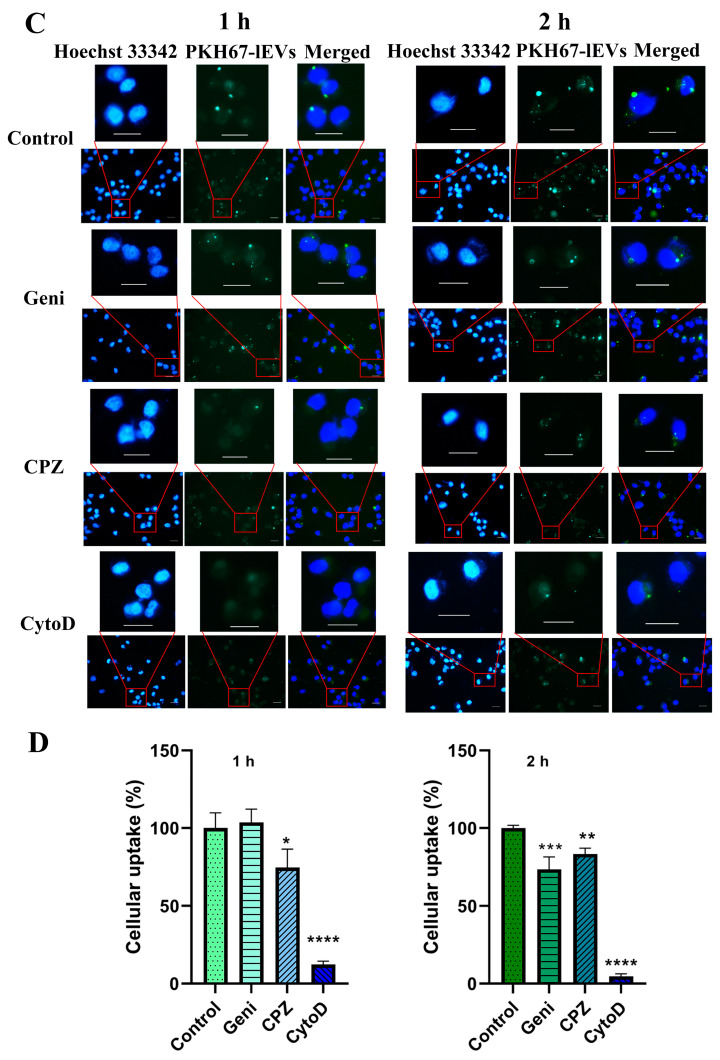
Representative fluorescence images of MIA PaCa-2 cells treated with MIA PaCa-2 cell-derived sEVs (**A**) or lEVs (**C**) for 1 h and 2 h in the presence of genistein (50 μg/mL), CPZ (10 μg/mL), or CytoD (10 μg/mL). Representative cells within the red boxed area are displayed in the upper panels for clarity. The cellular uptake (% of control) was measured by Fiji software (**B**,**D**) (data are presented as means ± SD, *n* = 3 cells in each case). Cells without pre-treatment with inhibitors were used as controls. * *p* < 0.05, ** *p* < 0.01, *** *p* < 0.005, **** *p* < 0.001. Scale bars: 20 μm. Images of the control groups were also used in Figure 5 as they were captured at the same time as those from the other groups.

Microscopic observation (Figure 7) shows that the cellular uptake of sEVs and lEVs was inhibited by all the inhibitors but to different degrees. At 1 h, the cellular uptake was significantly decreased to 41.0 ± 10.4% (*p* < 0.001) for sEVs and no change was found in lEVs when inhibiting caveolin-mediated endocytosis. In addition, the cellular uptake was reduced to 61.6 ± 5.9% (*p* < 0.01) and 74.6 ± 11.9% (*p* < 0.05) for sEVs and lEVs, respectively, when clathrin-mediated endocytosis was inhibited. These findings demonstrated that clathrin- and caveolin-mediated endocytosis both played major roles for sEVs’ cell entry, while lEVs predominantly used the clathrin-mediated pathway. Besides, in the presence of CytoD, the cellular uptake was reduced to 15.0 ± 11.2% and 12.3 ± 2.1% for sEVs and lEVs, respectively (*p* < 0.001), indicating that actin-dependent phagocytosis or macropinocytosis was the major cell entry pathway. It is worth noting that the high cellular uptake reduction by CytoD does not mean that actin-dependent endocytosis was the predominant pathway. A large vesicle (e.g., 500 nm in diameter) can carry 40 times more FI than a small vesicle of 80 nm in diameter in their membranes based on EV surface areas. This was supported by NTA results as well, which showed that only 1% of the sEVs and 3% of the lEVs were over 500 nm. Another reason could be that EVs might aggregate during the uptake process, leading to actin-dependent phagocytosis or macropinocytosis endocytosis. 

At 2 h, when caveolin-mediated endocytosis or clathrin-mediated endocytosis was inhibited, the cellular uptake of lEVs was reduced to 73.5 ± 8.0% (*p* < 0.005) and 83.3 ± 3.8% (*p* < 0.01), respectively. However, these inhibition effects were not observed with sEVs (96.3 ± 16.4% and 111.1 ± 11.3%, respectively). This may be due to the saturation of these pathways in sEVs. Another reason could be that more and more large vesicles entered into cells which caused high FI. While the cellular uptake of sEVs was similar at 1 h either in the presence or the absence of CytoD (16.2 ± 0.2% vs. 15.0 ± 11.2%). However, uptake of lEVs was further reduced by CytoD to 4.6 ± 1.7% from 12.3 ± 2.1% at 1 h (*p* < 0.001). Interestingly, some large dots attached to the cell membrane were shown in lEVs treated cells. To further explore the possible reason, natural sEVs and lEVs before adding to cells were observed by fluorescence microscopy (Supplementary Figure S1). The images showed that lEVs might aggregate easier than sEVs during the cell treatment. The agglomeration behaviour of particles might have an impact on interactions and subsequent cellular responses [64]. 

In summary, multiple endocytic pathways were responsible for cellular uptake of sEVs and lEVs. Caveolin- and clathrin-mediated endocytosis were more involved in sEVs cellular uptake than in lEVs. Tian et al. also supported the role of the clathrin-mediated pathway in PC12 cell-derived EVs (exosomes, 40–100 nm) uptake on their home cells [31], although some earlier studies pointed out that exosomes (121  ±  87 nm) are internalized into their home cells (mantle cell lymphoma) by the non-clathrin, non-caveolin1 pathway [32]. The comparison of these different results is complicated by the fact that different EV donor cell types and recipient cell types were studied [14,24]. 

**Figure 8:**
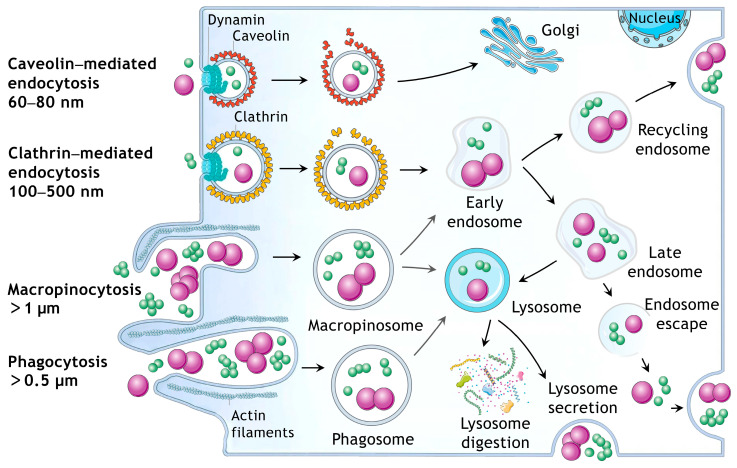
Schematic representation of the pathways of cellular internalisation of EVs based on their size. This includes phagocytosis, macropinocytosis, clathrin-mediated endocytosis, and caveolin-mediated endocytosis.

EVs’ internalization pathways have crucial implications for intracellular delivery of the payload. Vesicles endocytosed through caveolin-mediated pathway usually do not enter endosomes and are directly transferred to the Golgi [23] (Figure 8), while vesicles internalized by clathrin-mediated endocytosis are trafficked through endosomes-lysosomes followed by exocytosis or the degradation of the cargoes/drugs in the lysosomes [65]. Macropinocytosis and phagocytosis are both efficient in entering cells for small or large vesicles. However, by formation of macropinosomes or phagosomes, the vesicles are processed via the endosome–lysosome pathways [66,67]. Our study showed that about 40% of the sEVs and fewer lEVs internalized cells through caveolin-mediated endocytosis; therefore, sEVs can target to Golgi and release cargoes to the lysotol due to the endo-lysosome entrapment. The majority of lEVs may also be trafficked from macropinosomes or phagosomes to lysosomes following actin-dependent phagocytosis and macropinocytosis. Previously, it was reported that only 20–30% of the internalized sEVs release their cargoes to the cytosol with 24 h with the rest being entrapped or degraded in endo-lysosomes or re-secreted through the recycling machinery [68]. Interestingly, exocytosis of vesicles from cells have been recently exploited as a strategy to traverse cellular barriers in order to achieve tissue penetration in drug delivery [69], while the tendency for endosome/lysosome entrapment of EVs may be utilized to facilitate the treatment of lysosomal storage disease [69]. 

However, for those cargoes whose action sites are other subcellular localizations in the cytoplasm, the entrapment of EVs in endo/lysosomes could compromise their intracellular drug delivery efficiency. To avoid the entrapment, the endosome escape capability is a prerequisite [70]. pH-sensitive liposomes are well known for being able to undergo endosome escape. In our previous study, sEVs were successfully hybridized with pH-sensitive liposomes, thereby contributing to the endosome escape and providing three-times-higher drug delivery efficiency than the natural sEVs [13]. 

One of the limitations of this study lies in pre-treating the cells with pharmacological inhibitors to block specific endocytic pathways: (1) when the given endocytic pathway is inhibited, another can be activated to compensate, which is known as the cross-regulation effect [17]; (2) CytoD cannot distinguish between phagocytosis and macropinocytosis since phagosomes and macropinosomes are all coated with actin [71], making it impossible to identify the endocytic pathway precisely. Despite these challenges, this approach remains the most frequently used for this type of study [14,20].

## 4. Conclusions

This study demonstrated that the Integra CELLine culture system produced sEVs and lEVs much more efficiently than conventional flasks in the terms of the particle number as well as protein content of the EVs. The sEVs and lEVs produced by the Integra CELLine culture system entered MIA PaCa-2 cells via multiple pathways, including caveolin-mediated endocytosis, clathrin-mediated endocytosis, and actin-dependent phagocytosis or macropinocytosis. Caveolin- and clathrin-mediated endocytosis were the predominant pathways for sEVs compared to lEVs. Furthermore, both EV types were found to enter and become entrapped in endo-lysosomes. However, the complexity of cellular uptake highlights the necessity of investigating the endocytic mechanisms using different EV donor and recipient cell types to better understand these endocytic processes. 

## Figures and Tables

**Table 1:** List of inhibitors used in this study.

Inhibitor/Structure	Pathway	Mechanism of Activity	Concentrations
Genistein 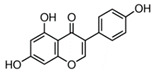	Caveolin-mediated endocytosis	A tyrosine kinase inhibitor, blocks the phosphorylation of caveolin-1	50 μg/mL [56] 75 nM [57]
Chlorpromazine (CPZ) 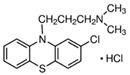	Clathrin-mediated endocytosis	A cationic amphiphilic drug, inhibits clathrin-coated pit formation by relocating clathrin and its adapter proteins from the plasma membrane to the endosomes	10 μg/mL [58,59] 1 μg/mL [60] 1 μM [61]
Cytochalasin D (CytoD) 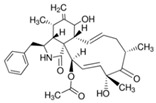	Actin-dependent endocytosis, i.e., phagocytosis and macropinocytosis	Block the actin polymerisation by occupying a faster-growing “barbed” end of actin filaments	10 μg/mL [36] 1 μg/mL [62] 20 μM [63]

## Data Availability

The original contributions presented in the study are included in the article/Supplementary Material, further inquiries can be directed to the corresponding author.

## Supplementary Materials

The following supporting information can be downloaded at: https://www.mdpi.com/article/10.3390/pharmaceutics16091206/s1. Figure S1: Natural sEVs and lEVs images under a fluorescence microscope, scale bars: 20 μm. The images showed that natural sEVs and lEVs had no difference in size before adding to cells, indicating lEVs might aggregate during the treatment.
