# Supplementary material for: Characterizing Extracellular Vesicles Generated from the Integra CELLine Culture System and Their Endocytic Pathways for Intracellular Drug Delivery
Source: Pharmaceutics. 2024 Sep 13;16(9):1206. doi: 10.3390/pharmaceutics16091206 (PMC11434853; doi:10.3390/pharmaceutics16091206)
Supplement: Supplementary file 1 [file pharmaceutics-16-01206-s001.zip › pharmaceutics-3140724-supplementary.pdf]

pharmaceutics

Supplementary Information

Understanding the endocytic pathways of extracellular vesicles produced by the Integra CELLine culture system for intracellular delivery

Tianjiao Geng[1,2], Lei Tian[1], Song Yee Paek[3], Euphemia Leung[4], Lawrence W. Chamley[3], Zimei Wu[1*]

[1] School of Pharmacy, Faculty of Medical and Health Sciences, University of Auckland, New Zealand
[2] Department of Pharmacy, Renji Hospital, School of Medicine, Shanghai Jiaotong University, Shanghai, China
[3] Department of Obstetrics and Gynaecology and Hub for Extracellular Vesicles Investigations; Faculty of Medical and Health Sciences, University of Auckland, New Zealand
[4] Auckland Cancer Society Research Centre; Faculty of Medical and Health Sciences, University of Auckland, New Zealand

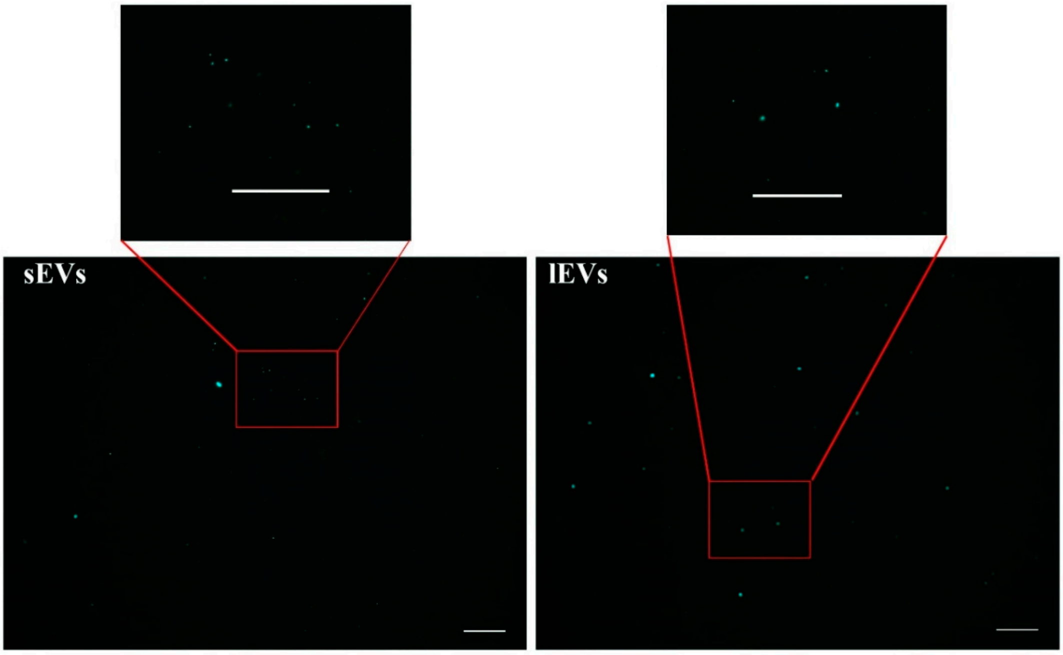

Figure S1. Natural sEVs and lEVs images under a fluorescence microscope, scale bars: 20 µm. The images showed that natural sEVs and lEVs had no difference in size before adding to cells, indicating lEVs might aggregate during the treatment.
